# "Healthy Men" and High Mortality: Contributions from a Population-Based Study for the Gender Paradox Discussion

**DOI:** 10.1371/journal.pone.0144520

**Published:** 2015-12-07

**Authors:** Tássia Fraga Bastos, Ana Maria Canesqui, Marilisa Berti de Azevedo Barros

**Affiliations:** Department of Public Health, School of Medical Sciences, State University of Campinas, Campinas, São Paulo, Brazil; National Institute of Health, ITALY

## Abstract

**Background:**

Inequalities between men and women in morbidity and mortality show a contrast, which has been called gender paradox. Most studies evaluating this paradox were conducted in high-income countries and, until now, few investigations have been performed in Brazil. This study aims to estimate the magnitude of inequalities between adult men and women in several dimensions: demographic and socioeconomic, health behaviors, morbidity, use of health services and mortality.

**Methods:**

The data were obtained from population-based household survey carried out in Campinas (Campinas Health Survey 2008/09) corresponding to 957 people, and data from the Mortality Information System (MIS) between 2009 and 2011. Prevalences and prevalence ratios were analyzed in order to verify the differences between men and women regarding socioeconomic and demographic variables, health behaviors, morbidities and consultations in the last two weeks. Mortality rates and the ratio between coefficients considering the underlying causes of death were calculated.

**Results:**

Women had a greater disadvantage in socioeconomic indicators, chronic diseases diagnosed by a health professional and referred health problems as well as make more use of health services, while men presented higher frequency of most unhealthy behaviors and excessive mortality for all causes investigated.

**Conclusions:**

The findings contribute to the discussion of gender paradox and demonstrate the need to employ health actions that consider the differences between men and women in the various health dimensions analyzed. The premature male mortality from preventable causes was outstanding, making clear the need for more effective prevention and health promotion directed to this segment of the population.

## Introduction

The higher prevalence of morbidity in women and, on the other hand, the higher male mortality rate is a contrast that it was first investigated in 1975 by Nathanson[1] and has been evidenced in several health research called *gender paradox*.[2,3,4]

It is noteworthy that men have higher mortality rates at all ages and by the main groups of causes of death (cancer, diseases of the circulatory, respiratory and digestive tract, and external causes) when compared to women.[5,6,7,8] The World Health Organization recognizes that a significant portion of deaths from noncommunicable chronic diseases (NCDs) worldwide occurs before 60 years old, reaching the percentage of 32.2% in men and 25.4% in women.[6] Excessive male mortality implies distinct life expectancies for men and women across the world, reaching a global gap of 5.7 years in 2010, ranging from 3.0 years in Western sub-Saharan Africa to 11.2 years in Eastern Europe.[9] In Brazil, the difference was 7.6 years (69.4 years for man and 77.0 years for women) in 2010.[9]

Overall, the studies indicate poorer health in women, such as self-rated health, physical illness, mental health and disabilities [10,11,12,13]. However, the finding of poorer health in women does not seem so clear, since, according review conducted by Oksuzyan[14], depends on the definition, severity and of the trajectory of the disease. Furthermore, the gender gap in morbidity and mortality vary over time and across places[15].

Among the possible explanations of the paradox are differentials between the sexes in biological risk, in health behaviors and social roles, in perception and behavior in facing disease and in the access to health services and treatments.[2,3,4,10].

Most of the studies on the *gender paradox* are conducted in high-income countries and focus on gender differences in life expectancy and mortality in middle-aged or older adults. There are no Brazilian studies evaluating the inequalities between adults men and women in different dimensions of health using jointly data from population-based health surveys and Mortality Information System seeking to analyze the *gender paradox* in a given population. The purpose of this article is to identify and discuss the possible explanations of the *gender paradox* by analyzing the inequalities between men and women in the prevalence of demographic and socioeconomic conditions, unhealthy behaviors, morbidities and health problems, consultations in the last two weeks and in mortality rates of the population of adults living in Campinas, Brazil.

## Methods

Campinas is a large-sized city, located in the state of São Paulo, in the southeastern of Brazil (Latitude: 22° 54 '20 "S/Longitude: 47 03 '39 "W), occupying an area of 794.6 Km^2^. In 2010, the population was 1,080.113 inhabitants, from which 98.3% live in the urban area. The Human Development Index (HDI) was 0.805 in 2010[16].

The data on socioeconomic and demographic conditions, unhealthy behaviors and health status was obtained from the Campinas Health Survey (ISACAMP), a population-based cross-sectional study carried out in the urban area of Campinas in 2008/2009. Data collection was made through a questionnaire, structured in thematic blocks that included: morbidities, accidents and violence episodes, emotional health, health-related behaviors, quality of life, use of health services, preventive practices, medication use and socioeconomic characteristics. The questionnaire used in the survey can be found at link: http://www.fcm.unicamp.br/fcm/sites/default/files/questionario_ingles.pdf.

The population that participated in the survey was obtained by probabilistic sampling, carried out in two stages. First, 50 census tracts of the urban area of Campinas were selected with probability proportional to the number of households, followed by a field survey to list all the private households of the selected tracts. In the next stage, households were drawn, aiming to get the sample size defined for three population subgroups: adolescents (10–19 years), adults (20–59 years) and elderly (60 years or older), who composed the domains of the study. Equal sized samples of 1,000 people for each one of these domains were drawn. With this number of interviews is possible to estimate proportions of 0.50 with sampling error between 4 and 5 percentage points with a confidence level of 95% and considering a design effect of 2.[17]

For the present study, the analyzed data refers to the adults’ domain. The variables analyzed in this study are listed below:

Demographic and socioeconomic: age (20–29, 30–39, 40–49, 50–59 years); skin color (white, nonwhite); religion (catholic, evangelical, others, no religion); marital status (married, living together, divorced/separated, single); schooling (in years); work status (working, unemployed, retired/pensioner, housewife, student/others); monthly per capita family income (in minimum wages); private health insurance (yes, no).

Health behaviors: alcohol abuse, measured by the Alcohol Use Disorders Identification Test—AUDIT, with 8 or more points being considered positive for the abuse; current smoker, regardless of the number of cigarettes, frequency and duration of habit; passive smoker (nonsmoker exposed to cigarette smoke for at least 1 hour per day); inactive in leisure physical activity: those who responded negatively to the question "*Do you practice regularly*, *at least once a week*, *some sort of physical exercise or sport*?"; frequency of consumption of fruits, vegetables and milk in less than four days a week; soft drinks and artificial juices in four or more days a week.

Health status: poor self-rated health (bad or very bad); common mental disorder (CMD) assessed by the Self-Reporting Questionnaire (SRQ-20), considering the presence of CMD when the score reached a value greater than or equal to 7; morbidities in the last two weeks prior to the interview; overweight (25 to <30 kg/m^2^) and obesity (≥ 30 kg/m^2^), measured by Body Mass Index (BMI), from reported information on weight and height; chronic diseases diagnosed by a health professional (obtained from a *checklist*): hypertension, diabetes, heart diseases, arthritis/ rheumatism/ osteoarthritis, asthma/ bronchitis/ emphysema, tendinitis/ repetitive strain injury (RSI)/ work-related musculoskeletal disorders (WRMD) and circulatory problems; presence of self-reported health problems present in a checklist: frequent headaches or migraines, back pain/spinal problems, allergies, emotional problems, dizziness or vertigo and insomnia.

Use of health services: consultations in the last two weeks (with physicians or other health professionals).

Death data from the residents of Campinas, between 2009 and 2011, were obtained from the Mortality Information System (SIM) and used for the analysis of the mortality profile according to sex. The estimated population for 2010 was obtained from the Health Information System—Tabnet/Campinas.[18] Age, sex and cause of death were the variables analyzed for the deaths.

### Data analysis

Prevalences and 95% confidence intervals of the variables were estimated. In the analysis of associations, we used the chi-square test with statistical significance level of 5%, and the crude and adjusted prevalence ratios were estimated with their respective 95% confidence intervals using the *Poisson* regression model. The prevalence ratios were adjusted for age and years of schooling to control for confounding. Data were analyzed using *Stata software* 11.0, which allows for considering the different weights of individuals in the sample, as well as the sample design.

Mortality rates were calculated for each sex and age groups using the average number of deaths in the three-year period 2009 to 2011 as numerator, and the population of 2010 referent to each age group and sex in the denominator. The mortality rates for the five leading groups of underlying causes of death (International Classification of Diseases, version 10, ICD-10) and for selected specific causes (ICD-BR-10) were calculated. Mortality rate ratios (MR) between men and women were also calculated to verify the magnitude of inequalities between the sexes.

This study has been approved by the Ethics Committee of the School of Medical Sciences of the University of Campinas (Permit number: 079/2007. The written informed consent was obtained from each participant prior to the interview.

## Results

The studied adult population (20–59 years of age) is composed of men with a mean age of 37.0 years (CI_95%_: 36.0–37.9) and women with 37.9 years old in average (CI_95%_: 36.9–38.9).

The analysis of demographic and socioeconomic conditions showed statistically significant differences between the sexes in age, religion, marital status, number of years of schooling, occupation and per capita income (Table 1). Much of the adult population was Catholic, but it was observed predominance of women in evangelical religion (35.5%), and a predominance of men among those who have no religion (17.2%). Concerning marital status, most adults were married, but there was a higher percentage of women among separated/judiciously separated while men stood out among singles. Women had a lower level of education and lower per capita income than men. In terms of occupation, 85.4% of men and 60.8% of women had some paid work (Table 1).

**Table 1:** Socioeconomic and demographic characteristics of adults, according to sex. Campinas, SP, Brazil-2008/09.

Variables	Men		Women		p
	n	%	n	%	
**Age (years)**					**0.0482**
20–29	155	35.2	150	29.8	
30–39	101	22.9	131	26.2	
40–49	98	21.2	126	24.5	
50–59	95	20.7	101	19.5	
**Skin color**					0.9969
White	327	73.3	370	73.3	
Nonwhite	120	26.7	138	26.7	
**Religion**					**0.0002**
Catholic	220	49.1	251	49.6	
Evangelical	131	29.0	183	35.5	
Others	20	4.6	33	6.8	
No religion	76	17.2	41	8.1	
**Marital status**					**0.0034**
Married	208	46.0	236	46.3	
Living together	72	16.0	78	15.3	
Divorced/separated	36	7.9	69	13.3	
Single	133	30.1	125	25.0	
**Schooling (in years)**					**0.0214**
0–8	166	35.7	219	41.9	
9–11	149	33.0	150	29.4	
12 or more	134	31.3	139	28.7	
**Work status**					**<0.0001**
Working	383	85.4	307	60.8	
Unemployed	33	7.2	27	5.4	
Retired/pensioner	22	4.7	19	3.7	
Housewife	0	0	140	27.1	
Student/others	11	2.7	15	3.1	
**Monthly *per capita* family income (in minimum wages)**					**0.0151**
≤1	171	37.4	226	43.7	
1–3	194	43.0	186	36.4	
>3	84	19.6	96	20.0	
**Private health insurance**					0.3713
Yes	189	43.0	223	45.0	
No	260	57.0	285	55.0	

As for health-related behaviors, there was a lower frequency of consumption of fruits, vegetables and milk and higher intake of soft drinks among men. The alcohol abuse was 4.8 times and smoking 1.5 times more prevalent in men. Among the health behaviors, only inactivity in physical activity in leisure context was higher among women (Table 2).

**Table 2:** Prevalence and prevalence ratios of health behaviors of adults, according to sex. Campinas, SP, Brazil-2008/09.

Variables	Prevalence				p	Crude PR	Adjusted PR*
	Men		Women				
	N	%	N	%			
**Alcohol abuse**	71	15.9	17	3.4	**<0.0001**	**0.21 (0.11–0.42)**	**0.21 (0.10–0.42)**
**Current smoker**	112	24.8	85	16.7	**0.0021**	**0.67 (0.53–0.86)**	**0.65 (0.50–0.83)**
**Passive smoker**	63	19.2	86	20.3	0.6340	1.06 (0.82–1.37)	1.06 (0.83–1.36)
**Inactive in leisure time physical activity**	260	57.5	384	75.1	**<0.0001**	**1.31 (1.17–1.46)**	**1.28 (1.15–1.44)**
**Fruits consumption <4 days a week**	243	54.2	203	39.6	**<0.0001**	**0.73 (0.65–0.82)**	**0.73 (0.64–0.82)**
**Vegetables consumption <4 days a week**	141	31.3	135	26.6	**0.0434**	**0.85 (0.73–0.99)**	**0.84 (0.71–0.99)**
**Milk consumption <4 days a week**	202	45.1	195	38.2	**0.0395**	**0.85 (0.72–0.99)**	**0.84 (0.72–0.99)**
**Soft drinks and artificial juices consumption ≥ 4 days a week**	159	35.5	135	26.4	**0.0053**	**0.74 (0.60–0.92)**	**0.74 (0.60–0.92)**

*Prevalence ratio (PR) adjusted for age and schooling, considering men as the reference category.

In the dimension of health status, sex was observed to be strongly associated with most events, even after adjustment for age and level of education. The prevalence of common mental disorder was 2.28 in women compared to men, and morbidity in the last two weeks was 47% higher in women than men. Women also showed more frequently presence of at least one diagnosed chronic disease, with higher prevalence of hypertension (PR = 1.34), arthritis/ rheumatism/ osteoarthritis (PR = 4.84) and circulatory problems (PR = 2.19). There were no statistically significant differences between the sexes for the others chronic diseases investigated. In the array of health problems, all had higher prevalence in women, especially, with greater magnitude of association, emotional problems (PR = 2.17), dizziness/ vertigo (PR = 2.23) and insomnia (PR = 1.99). Regarding the use of health services, women reported prevalence 46% greater of consultation with a health professional (Table 3).

**Table 3:** Prevalence and prevalence ratio for health conditions and use of health services of adults, according to sex. Campinas, SP, Brazil-2008/09.

Variables	Prevalence						
	Men		Women				
	n	%	n	%	p	Crude PR	Adjusted PR*
**Poor self-rated health**	26	5.6	39	7.5	0.2898	1.33 (0.77–2.29)	1.20 (0.69–2.09)
**Common Mental Disorders (SRQ-20)**	27	6.0	75	14.7	**<0.0001**	**2.43 (1.60–3.70)**	**2.28 (1.51–3.46)**
**Morbidity in the last two weeks**	66	14.7	111	21.9	**0.0029**	**1.49 (1.15–1.93)**	**1.47 (1.13–1.91)**
**Overweight**	154	34.2	147	28.6	0.1035	0.84 (0.68–1.04)	0.82 (0.66–1.01)
**Obesity**	69	15.5	95	18.4	0.2148	1.19 (0.90–1.59)	1.16 (0.87–1.54)
**One or more chronic diseases**	141	31.4	211	41.8	**0.0033**	**1.33 (1.11–1.60)**	**1.28 (1.08–1.53)**
**Hypertension**	53	11.5	86	16.5	**0.0138**	**1.43 (1.07–1.90)**	**1.34 (1.02–1.75)**
**Diabetes**	13	2.8	24	4.7	0.0952	1.65 (0.91–3.00)	1.52 (0.81–2.85)
**Heart diseases**	13	2.9	22	4.2	0.2735	1.46 (0.73–2.91)	1.38 (0.71–2.69)
**Arthritis/ rheumatism/ osteoarthritis**	5	1.1	30	5.8	**0.0002**	**5.16 (2.08–12.76)**	**4.84 (1.92–12.21)**
**Asthma/ bronchitis/ emphysema**	12	2.7	21	4.1	0.2563	1.54 (0.72–3.31)	1.53 (0.71–3.28)
**Tendinitis/RSI/ WRMD**	20	4.6	38	7.5	0.1117	1.64 (0.88–3.06)	1.63 (0.87–3.06)
**Circulatory problems**	24	5.2	65	12.5	**<0.0001**	**2.39 (1.65–3.48)**	**2.19 (1.49–3.22)**
**One or more health problems**	272	60.2	398	78.1	**<0.0001**	**1.30 (1.20–1.40)**	**1.28 (1.19–1.38)**
**Frequent headaches/migraines**	94	20.6	179	34.7	**<0.0001**	**1.68 (1.35–2.10)**	**1.63 (1.30–2.05)**
**Back pain/spinal problems**	123	27.0	180	35.1	**0.0063**	**1.30 (1.08–1.56)**	**1.25 (1.04–1.49)**
**Allergies**	102	23.0	165	32.5	**0.0024**	**1.41 (1.13–1.76)**	**1.44 (1.16–1.79)**
**Emotional problems**	51	11.5	130	25.6	**<0.0001**	**2.23 (1.61–3.08)**	**2.17 (1.58–2.98)**
**Dizziness/vertigo**	24	5.3	65	12.6	**0.0009**	**2.38 (1.42–3.99)**	**2.23 (1.34–3.72)**
**Insomnia**	43	9.6	102	19.9	**<0.0001**	**2.07 (1.49–2.86)**	**1.99 (1.43–2.78)**
**Consultation in the last two weeks**	62	14.0	112	22.2	**0.0007**	**1.59 (1.22–2.06)**	**1.46 (1.14–1.88)** **

*Prevalence ratio (PR) adjusted for age and education, considering men as the reference category.

**Prevalence ratio (PR) adjusted for age, education and number of chronic diseases.

Concerning to mortality, the rates were more than two times superior in men in all age groups, ranging from 3.4 in the groups of 20 to 29 years old, to 2.2 in the segment of 50 to 59 years old. Considering the five top groups of the underlying causes of death, men had a higher risk of dying in all of them, achieving a 6.4 times greater risk of dying from external causes of morbidity and mortality. Among the specific causes of death, the greatest differences between the sexes were found in deaths due to homicides (MR = 9.0), transport accidents (MR = 6.6), fibrosis and cirrhosis of the liver (MR = 6.7) and pneumonia (MR = 3.1). Except for hypertension and cerebrovascular diseases, in all other specific causes, men had coefficients superior to two times when compared to those of the women (Table 4).

**Table 4:** Mortality rates* and mortality ratios by age and underlying causes of death (ICD-10 and ICD-BR-10), according to sex. Campinas, SP, Brazil, 2009–2011.

Variables	Mortality rates		Mortality ratios (1)/(2)
	Men^(1)^	Women^(2)^	
**Age**			
20–29	1.7	0.5	3.4
30–39	2.3	1.0	2.4
40–49	4.5	1.9	2.4
50–59	9.2	4.2	2.2
Total	3.9	1.7	2.3
**Groups of underlying causes (ICD-10)**			
II. Neoplasms (tumors)	60.1	51.5	1.2
IX. Circulatory diseases	86.7	41.6	2.1
X. Respiratory diseases	31.9	14.0	2.3
XI. Digestive tract diseases	33.7	10.4	3.2
XX. External causes	109.5	17.2	6.4
**Causes (ICD-BR-10)**			
Malignant neoplasm of stomach	5.9	2.3	2.6
Malignant neoplasm of trachea, bronchus and lungs	6.9	3.2	2.1
Diabetes mellitus	7.3	3.3	2.2
Hypertensive diseases	4.6	3.0	1.5
Acute myocardial infarction	44.5	18.2	2.5
Cerebrovascular diseases	17.5	10.1	1.7
Pneumonia	18.6	6.0	3.1
Liver fibrosis and cirrhosis	7.9	1.2	6.7
Transport accidents	40.3	6.1	6.6
Homicide	40.4	4.5	9.0

* deaths per 100,000 inhabitants

## Discussion

The results of the study revealed inequalities between men and women in all dimensions analyzed. Women had lower levels of education and income and entry into the labor market than men. While men showed, in general, higher frequency of unhealthy habits, women reported more health problems, with remarkable inequality in arthritis, circulatory problems, common mental disorders and emotional problems. However, the highest mortality rates are found in the male population in the main groups of causes of death and in all age groups analyzed, confirming the *gender paradox*.

In the dimension of health-related behaviors, corroborating the national[19,20] and abroad[21] literature, men showed greater disadvantage compared to women regarding alcohol abuse. A previous research undertaken in 2003 in Campinas found that prevalence of alcohol abuse among men was 3,53 times higher than for women,[22] as in the present study.

The magnitude of the prevalence ratio of smokers between men and women in Campinas (PR = 1.5) resembles the reality found in the Brazilian capitals in 2008 (PR = 1.6).[23] Higher prevalence of smoking have also been found in men in all 14 low and middle income countries investigated by the *Global Adult Tobacco Survey* (GATS), which identified crude prevalence ratios ranging from 1.6 in Uruguay to 75.0 in Egypt.[24]

Regarding physical activity in leisure context, the inactivity was higher among women. This behavior was also observed in other Brazilian studies.[25,26,27] There were statistically significant gender differences in European Union countries, favoring men in 15 out of 27 analyzed countries.[28] According to the authors, in countries where there is greater gender equality (measured by international indicators *United Nations Development Programme's Gender Empowerment Measure—GEM and World Economic Forum's Gender Gap Index*—GGI), differences in physical activity in leisure context are inexistent.

There were also inequalities between the sexes, although with lower prevalence ratios, in food intake. Men reported less frequently intake of fruits, vegetables and milk lessand more frequently of soft drinks. Likewise in Campinas, gender inequalities were observed in the consumption of these foods in the Brazilian population in 2006,[29] and 2012,[30] with consumption of poorer quality among men.

A review of anthropological research on the practices and preferences of food consumption among low-income population segments detected the lower appreciation for consumption of fruits and vegetables, classified as "weak" or "soft" foods due to the fact that these do not satisfy the sensation of hunger and do not offer the body strength and energy needed to work.[31,32] There is a trend for men showing a lower preference for these foods than women.

A variety of health indicators was used to verify gender differences in the dimension of morbidities. Likewise in this study, the National Research by Household Sampling, held in 2008,[33] found higher prevalences in women in the majority of the chronic diseases analyzed. Literature show that, in general, women have lower socioeconomic status (as seen in this article) and high exposure to social stressors associated with the emergence of non-fatal acute and chronic conditions, resulting in a greater proportion of morbidity in relation to men.[4,10,11]

The largest morbidity prevalence referred by women may also be related to their greater access to health services, as found in this study and in national[34] and international[3,10] health surveys. In general, men have a lower demand for health services and some of the factors identified by other studies were the outpatient appointment hours coincide with their working hours and due to the way they deal with their health and well-being, influenced by beliefs reflected in their health behavior.[34,35,36] Given this reality, it is possible that the prevalence of chronic morbidities referred, diagnosed by a health professional, are underestimated by men. However, in the case of health problems or referred symptoms with no need for medical diagnosis, men tend to have them or report them less often than women, supporting gender studies that highlight the difficulty of man to recognize health problems.[5]

The lower report of morbidities is not indicative of good health, as illustrated by the fact that men have the highest mortality rates, especially at early ages, evidenced by the higher male mortality in all age groups and in the major groups of underlying cause of death, as seen in this study and in studies that investigated the mortality profile in Brazil.[8,37] In addition to deaths from accidents and violence, men have higher rates than those of women into several groups of chronic diseases. These deaths have multiple factors associated with their causes, including, with great relevance, the unhealthy behaviors (smoking, alcohol abuse, and worse food consumption), adopted by men in higher proportion.

A study that evaluated the magnitude of gender differences in mortality from all causes in 30 European countries found that 40–60% of the differences in mortality between men and women are attributable to smoking, whereas alcohol consumption contributes to 10–20% of these differences.[38] It is noteworthy that in the present study these two factors had the highest differences between sexes in the dimension of behaviors investigated.

The differences between men and women in morbidity and mortality found in this research confirm the *gender paradox*. The detailed analysis of the differences between men and women in each of the dimensions of health, showed some of the possible explanations for this paradox. It was also observed a *gender paradox* between the dimensions of health behaviors and health status: women report healthier behaviors than man, but have more chronic diseases and health problems compared to them. So far we have not found Brazilian studies using together data from population-based health survey and mortality to measure inequalities in the various dimensions of health of adult men and women, aiming to contribute to the discussion of *gender paradox*.

In addition to the explanations for the paradox discussed so far, men and women have different relations with their own bodies, what is shaped by moral and aesthetic reasons allied to the constitution of femininity and masculinity patterns, turning more socially acceptable a greater attention dedicated by women to their bodies and their sensations than men, in which such practices would compromise the image of virility associated with their bodily behaviors and health care. Gender divisions also have repercussions on ways to endure the pain in silence, signaling virility in some cultures, while women value the explicit expression of their feelings.[39]

The observed differences between men and women should be considered in order to promote health actions equitably between these segments. Given the higher adoption of unhealthy behavior and lower demand for health services, by men, which may explain the lower presence of morbidities among them and the higher mortality rates, it is suggested that young men are still poorly supported by health services. Recognizing the men singularities in the context of morbidity and excess mortality, the National Policy for Integral Attention to Men's Health was launched in Brazil in 2009. In support of the policy, this study reinforces the importance of promoting strategies approaching men of health services, especially primary care, and investing in health education to stimulate self-care and changes in behaviors that may endanger their lives at an early age.

## Supporting Information

S1 Table Socioeconomic and demographic characteristics of adults, according to sex. Campinas, SP, Brazil-2008/09.
(DOCX)

S2 Table Prevalence and prevalence ratios of health behaviors of adults, according to sex. Campinas, SP, Brazil-2008/09.
(DOCX)

S3 Table Prevalence and prevalence ratio for health conditions and use of health services of adults, according to sex. Campinas, SP, Brazil-2008/09.
(DOCX)

S4 Table Mortality rates* and mortality ratios by age and underlying causes of death (ICD-10 and ICD-BR-10), according to sex. Campinas, SP, Brazil, 2009–2011.
(DOCX)

## References

[1] Nathanson CA. Illness and the feminine role: a theoretical review. Soc Sci Med, 1975 9: p. 57–62. 1093257 10.1016/0037-7856(75)90094-3

[2] Case A, Paxson CH. Sex differences in morbidity and mortality. Demography, 2005 42(2): p. 189–214. 15986983 10.1353/dem.2005.0011

[3] Perelman J, Fernandes A, Mateus C. Gender disparities in health and healthcare: results from the Portuguese National Health Interview Survey. Cad Saude Publica, 2012 28(12): p. 2339–2348 23288066 10.1590/s0102-311x2012001400012

[4] Read JG, Gorman BK. Gender and Health Inequality. Annu Rev Sociol, 2010 36: p. 371–386.

[5] Courtenay WH. Behavioral factors associated with disease, injury, and death among men: evidence and implications for prevention. J Mens Stud, 2000 9(1): p. 81–142.

[6] WHO. World Health Organization. Noncommunicable diseases country profiles 2011. Geneva: World Health Organization, 2011.

[7] Barros MBA, Marín-León L, Almeida SM, Restituti MC, Belon AP. Mortalidade e Gênero. Campinas: [s.n.], 2008. Boletim de Mortalidade n° 42.

[8] Laurenti R, Jorge MHPM, Goltlieb SLD. Epidemiological profile of men: morbidity and mortality. Cienc Saude Coletiva, 2005 10(1): p. 35–46.

[9] Wang H, Dwyer-Lindgren L, Lofgren KT, Rajaratnam JK, Marcus JR, Levin-Rector A et al Age-specific and sex-specifi c mortality in 187 countries, 1970–2010: a systematic analysis for the Global Burden of Disease Study 2010. Lancet, 2012 380: p. 2071–94. 10.1016/S0140-6736(12)61719-X 23245603

[10] Verbrugge LM. The Twain meet: empirial explanations of sex differences in health and mortality. J Health Soc Behav, 1989 30: p.282–304. 2778300

[11] Crimmins EM, Kim JK, Solé-Auró A. Gender differences in health: Results from SHARE, ELSA and HRS. Eur J Pub Health, 2010 21: p. 81–91. 20237171 10.1093/eurpub/ckq022 PMC3023013

[12] Lindahl-Jacobsen R, Hanson HA, Oksuzyan A, Mineau GP, Christensen K, Smith KR. The male-female health-survival paradox and sex differences in cohort life expectancy in Utah, Denmark, and Sweden 1850–1910. Ann Epidemiol, 2013 23: p. 161–166. 10.1016/j.annepidem.2013.02.001 23453386 PMC3651922

[13] Oksuzyan A, Shkolnikova M, Vaupel JW, Christensen K, Shkolnikov VM. Sex differences in health and mortality in Moscow and Denmark. Eur J Epidemiol, 2014 29(4): p. 243–252. 10.1007/s10654-014-9893-4 24668060 PMC4090601

[14] Oksuzyan A, Juel K, Vaupel JW, Christensen K. Men: Good health and high mortality. Sex differences in health and aging. Aging Clin Exp Res, 2008 20: p. 91–102. 18431075 10.1007/bf03324754 PMC3629373

[15] Oksuzyan A, Crimmins E, Saito Y, O’Rand A, Vaupel JW, Christensen K. Cross-national comparison of sex differences in health and mortality in Denmark, Japan and the US. Eur J Epidemiol, 2010 25: p. 471–480. 10.1007/s10654-010-9460-6 20495953 PMC2903692

[16] IBGE. Instituto Brasileiro de Geografia e Estatística [homepage na internet]. Demographic census 2010: population by municipality. 2010 [01/05/2012]; Available: http://www.ibge.gov.br/home/estatistica/populacao/censo2010/tabelas_pdf/total_populacao_sao_paulo.pdf.

[17] ISA-Campinas 2008–2009. Sampling plan. [05/12/2014]; Available:http://www.fcm.unicamp.br/fcm/sites/default/files/plano_de_amostragem.pdf.

[18] Campinas. Municipal Department of Health. Mortality Information System—TabNet. [05/12/2014]; Available: http://tabnet.campinas.sp.gov.br/dh?sim/sim.def.

[19] Ferreira LN, Sales ZN, Casotti CA, Bispo Júnior JP, Braga Júnior ACR. Alcohol consumption and associated factors in a city in Northeast Brazil. Cad Saude Publica, 2011 27(8): p. 1473–1486. 21876996 10.1590/s0102-311x2011000800003

[20] Freitas ICM, Moraes SA. Alcohol addiction and associated factors in adults in Ribeirão Preto, São Paulo State, Brazil, 2006: the OBEDIARP Project. Cad Saude Publica, 2011 27(10): p. 2021–2031. 22031206

[21] Redonnet B, Chollet A, Fombonne E, Bowes L, Melchior M. Tobacco, alcohol, cannabis and other illegal drug use among young adults: the socioeconomic context. Drug Alcohol Depen, 2012 121: p. 231–239. 10.1016/j.drugalcdep.2011.09.002 21955362

[22] Barros MBA, Botega NJ, Dalgalarrondo P, Marin-León L, Oliveira HB. Prevalence of alcohol abuse and associated factors in a population-based study. Rev Saude Publica, 2007 41: p. 502–509. 17589746 10.1590/s0034-89102006005000032

[23] Malta DC, Oliveira TP, Luz M, Stopa SR, Silva Junior JB, Reis AAC. Smoking trend indicators in Brazilian capitals, 2006–2013. Cienc Saude Col, 2015 20(3): p. 631–640. 10.1590/1413-81232015203.15232014 25760105

[24] Giovino GA, Mirza SA, Samet JM, Gupta PC, Jarvis MJ, Bhala N, et al Tobacco use in 3 billion individuals from 16 countries: an analysis of nationally representative cross-sectional household surveys. Lancet, 2012 380: p. 668–679. 10.1016/S0140-6736(12)61085-X 22901888

[25] Azevedo MR, Araújo CLP, Reichert FF, Siqueira FV, Silva MC, Hallal PC. Gender differences in leisure-time physical activity. Int J Public Health, 2007 52: p. 8–15. 17966815 10.1007/s00038-006-5062-1 PMC2778720

[26] Del Duca GF, Nahas MV, de Sousa TF, Mota J, Hallal PC, Peres KG. Clustering of physical inactivity in leisure, work, commuting and household domains among Brazilian adults. Public Health, 2013 127: p. 530–537. 10.1016/j.puhe.2013.02.013 23706706

[27] Zanchetta LM, Barros MBA, César CLG, Carandina L, Goldbaum M, Alves MCGP. Physical inactivity and associated factors in adults, São Paulo, Brazil. Rev Bras Epidemiol, 2010 13(3): p. 387–399. 20857026 10.1590/s1415-790x2010000300003

[28] Van Tuyckom C, Van de Velde S, Bracke P. Does country-context matter? A cross-national analysis of gender and leisure time physical inactivity in Europe. Eur J Public Health, 2013 23(3): p. 452–457 10.1093/eurpub/cks009 22383475

[29] Jaime PC, Figueiredo ICR, Moura EC, Malta DC. Factors associated with fruit and vegetable consumption in Brazil, 2006. Rev Saude Publica, 2009 43(Supl 2):57–64. 19936499 10.1590/s0034-89102009000900008

[30] Brasil. Ministério da Saúde. Vigitel Brasil 2012: vigilância de fatores de risco e proteção para doenças crônicas por inquérito telefônico / Ministério da Saúde, Secretaria de Vigilância em Saúde, Departamento de Vigilância de Doenças e Agravos não Transmissíveis e Promoção de Saúde.–Brasília: Ministério da Saúde, 2013.

[31] Canesqui AM. Comentários sobre os estudos antropológicos da alimentação In: Canesqui AM, Garcia RWD (org). Antropologia e Alimentação: um diálogo possível. Rio de Janeiro: Editora Fiocruz 2005; 23–47.

[32] Daneil JMP, Cravo VZ. Valor Social e Cultural da Alimentação In: Canesqui AM, Garcia RWD (org). Antropologia e Alimentação: um diálogo possível. Rio de Janeiro Editora Fiocruz 2005: 57–68.

[33] Barros MBA, Francisco PMSB, Zanchetta LM, Cesar CLG. Trends in social and demographic inequalities in the prevalence of chronic diseases in Brazil. PNAD: 2003–2008. Cienc Saude Coletiva, 2011 16(9): p. 3755–3768. 10.1590/s1413-81232011001000012 21987319

[34] Pinheiro RS, Viacava F, Travassos C, Brito AS. Gender, morbidity, access and utilization of health services in Brazil. Cienc Saude Coletiva, 2002 7(4): p. 687–707.

[35] Gomes R, Moreira MCN, Nascimento EF, Rebello LEFS, Couto MT, Schraiber LB. Men don't come! Absence and/or invisibility in primary healthcare services. Cienc Saude Coletiva, 2011 16(Supl. 1): p. 983–992 10.1590/s1413-81232011000700030 21503446

[36] White AK, Fawkner H, Holmes M. Is there a case for differential treatment of young men and women? Med J Aust, 2006 185(8): p. 454–5. 17137439 10.5694/j.1326-5377.2006.tb00647.x

[37] Stevens A, Schmidt MI, Duncan BB. Gender inequalities in non communicable disease mortalityin Brazil 2012. Ciênc Saúde Colet, 2012 17(10): p. 2627–2634. 10.1590/s1413-81232012001000012 23099750

[38] McCartney G, Mahmood L, Leyland AH, Batty GD, Hunt K. Contribution of smoking-related and alcohol-related deaths to the gender gap in mortality: evidence from 30 European countries. Tob Control, 2011 20: p. 166–168. 10.1136/tc.2010.037929 21228431 PMC3045524

[39] Sarti CA. A dor, o indivíduo e a cultura. Saude Soc, 2001 10(1): p. 3–13.

