# Supplementary material for: "Healthy Men" and High Mortality: Contributions from a Population-Based Study for the Gender Paradox Discussion
Source: PLoS One. 2015 Dec 7;10(12):e0144520. doi: 10.1371/journal.pone.0144520 (PMC4671596; doi:10.1371/journal.pone.0144520)
Supplement: S1 Table — Campinas, SP, Brazil-2008/09. (DOCX) [file pone.0144520.s001.docx]

**Table 1:** Socioeconomic and demographic characteristics of adults, according to sex. Campinas, SP, Brazil-2008/09.

| **Variables** | **Men** | | **Women** | | **p** |
| --- | --- | --- | --- | --- | --- |
|  | **n** | **%** | **n** | **%** |  |
| **Age (years)** |  |  |  |  | **0.0482** |
| 20-29 | 155 | 35.2 | 150 | 29.8 |  |
| 30-39 | 101 | 22.9 | 131 | 26.2 |  |
| 40-49 | 98 | 21.2 | 126 | 24.5 |  |
| 50-59 | 95 | 20.7 | 101 | 19.5 |  |
| **Skin color** |  |  |  |  | 0.9969 |
| White | 327 | 73.3 | 370 | 73.3 |  |
| Nonwhite | 120 | 26.7 | 138 | 26.7 |  |
| **Religion** |  |  |  |  | **0.0002** |
| Catholic | 220 | 49.1 | 251 | 49.6 |  |
| Evangelical | 131 | 29.0 | 183 | 35.5 |  |
| Others | 20 | 4.6 | 33 | 6.8 |  |
| No religion | 76 | 17.2 | 41 | 8.1 |  |
| **Marital status** |  |  |  |  | **0.0034** |
| Married | 208 | 46.0 | 236 | 46.3 |  |
| Living together | 72 | 16.0 | 78 | 15.3 |  |
| Divorced/separated | 36 | 7.9 | 69 | 13.3 |  |
| Single | 133 | 30.1 | 125 | 25.0 |  |
| **Schooling ( in years)** |  |  |  |  | **0.0214** |
| 0-8 | 166 | 35.7 | 219 | 41.9 |  |
| 9-11 | 149 | 33.0 | 150 | 29.4 |  |
| 12 or more | 134 | 31.3 | 139 | 28.7 |  |
| **Work status** |  |  |  |  | **<0.0001** |
| Working | 383 | 85.4 | 307 | 60.8 |  |
| Unemployed | 33 | 7.2 | 27 | 5.4 |  |
| Retired/pensioner | 22 | 4.7 | 19 | 3.7 |  |
| Housewife | 0 | 0 | 140 | 27.1 |  |
| Student/others | 11 | 2.7 | 15 | 3.1 |  |
| **Monthly *per capita* family income (in minimum wages)** |  |  |  |  | **0.0151** |
| **≤**1 | 171 | 37.4 | 226 | 43.7 |  |
| 1-3 | 194 | 43.0 | 186 | 36.4 |  |
| >3 | 84 | 19.6 | 96 | 20.0 |  |
| **Private health insurance** |  |  |  |  | 0.3713 |
| Yes | 189 | 43.0 | 223 | 45.0 |  |
| No | 260 | 57.0 | 285 | 55.0 |  |
