# Supplementary material for: "Healthy Men" and High Mortality: Contributions from a Population-Based Study for the Gender Paradox Discussion
Source: PLoS One. 2015 Dec 7;10(12):e0144520. doi: 10.1371/journal.pone.0144520 (PMC4671596; doi:10.1371/journal.pone.0144520)
Supplement: S3 Table — Campinas, SP, Brazil-2008/09. (DOCX) [file pone.0144520.s003.docx]

**Table 3:** Prevalence and prevalence ratio for health conditions and use of health services of adults, according to sex. Campinas, SP, Brazil-2008/09.

| **Variables** | **Prevalence** | | | |  |  |  |
| --- | --- | --- | --- | --- | --- | --- | --- |
|  | **Men** | | **Women** | |  | | |
|  | **n** | **%** | **n** | **%** | **p** | **Crude PR** | **Adjusted PR*** |
| **Poor self-rated health** | 26 | 5.6 | 39 | 7.5 | 0.2898 | 1.33 (0.77-2.29) | 1.20 (0.69-2.09) |
| **Common Mental Disorders (SRQ-20)** | 27 | 6.0 | 75 | 14.7 | **<0.0001** | **2.43 (1.60-3.70)** | **2.28 (1.51-3.46)** |
| **Morbidity in the last two weeks** | 66 | 14.7 | 111 | 21.9 | **0.0029** | **1.49 (1.15-1.93)** | **1.47 (1.13-1.91)** |
| **Overweight** | 154 | 34.2 | 147 | 28.6 | 0.1035 | 0.84 (0.68-1.04) | 0.82 (0.66-1.01) |
| **Obesity** | 69 | 15.5 | 95 | 18.4 | 0.2148 | 1.19 (0.90-1.59) | 1.16 (0.87-1.54) |
| **One or more chronic diseases** | 141 | 31.4 | 211 | 41.8 | **0.0033** | **1.33 (1.11-1.60)** | **1.28 (1.08-1.53)** |
| **Hypertension** | 53 | 11.5 | 86 | 16.5 | **0.0138** | **1.43 (1.07-1.90)** | **1.34 (1.02-1.75)** |
| **Diabetes** | 13 | 2.8 | 24 | 4.7 | 0.0952 | 1.65 (0.91-3.00) | 1.52 (0.81-2.85) |
| **Heart diseases** | 13 | 2.9 | 22 | 4.2 | 0.2735 | 1.46 (0.73-2.91) | 1.38 (0.71-2.69) |
| **Arthritis/ rheumatism/ osteoarthritis** | 5 | 1.1 | 30 | 5.8 | **0.0002** | **5.16 (2.08-12.76)** | **4.84 (1.92-12.21)** |
| **Asthma/ bronchitis/ emphysema** | 12 | 2.7 | 21 | 4.1 | 0.2563 | 1.54 (0.72-3.31) | 1.53 (0.71-3.28) |
| **Tendinitis/RSI/ WRMD** | 20 | 4.6 | 38 | 7.5 | 0.1117 | 1.64 (0.88-3.06) | 1.63 (0.87-3.06) |
| **Circulatory problems** | 24 | 5.2 | 65 | 12.5 | **<0.0001** | **2.39 (1.65-3.48)** | **2.19 (1.49-3.22)** |
| **One or more health problems** | 272 | 60.2 | 398 | 78.1 | **<0.0001** | **1.30 (1.20-1.40)** | **1.28 (1.19-1.38)** |
| **Frequent headaches/migraines** | 94 | 20.6 | 179 | 34.7 | **<0.0001** | **1.68 (1.35-2.10)** | **1.63 (1.30-2.05)** |
| **Back pain/spinal problems** | 123 | 27.0 | 180 | 35.1 | **0.0063** | **1.30 (1.08-1.56)** | **1.25 (1.04-1.49)** |
| **Allergies** | 102 | 23.0 | 165 | 32.5 | **0.0024** | **1.41 (1.13-1.76)** | **1.44 (1.16-1.79)** |
| **Emotional problems** | 51 | 11.5 | 130 | 25.6 | **<0.0001** | **2.23 (1.61-3.08)** | **2.17 (1.58-2.98)** |
| **Dizziness/vertigo** | 24 | 5.3 | 65 | 12.6 | **0.0009** | **2.38 (1.42-3.99)** | **2.23 (1.34-3.72)** |
| **Insomnia** | 43 | 9.6 | 102 | 19.9 | **<0.0001** | **2.07 (1.49-2.86)** | **1.99 (1.43-2.78)** |
| **Consultation in the last two weeks** | 62 | 14.0 | 112 | 22.2 | **0.0007** | **1.59 (1.22-2.06)** | **1.46 (1.14-1.88)**** |

*Prevalence ratio (PR) adjusted for age and education, considering men as the reference category.

**Prevalence ratio (PR) adjusted for age, education and number of chronic diseases.
