# Supplementary material for: "Healthy Men" and High Mortality: Contributions from a Population-Based Study for the Gender Paradox Discussion
Source: PLoS One. 2015 Dec 7;10(12):e0144520. doi: 10.1371/journal.pone.0144520 (PMC4671596; doi:10.1371/journal.pone.0144520)
Supplement: S4 Table — Campinas, SP, Brazil, 2009–2011. (DOCX) [file pone.0144520.s004.docx]

**Table 4.** Mortality rates* and mortality ratios by age and underlying causes of death (ICD-10 and ICD-BR-10), according to sex. Campinas, SP, Brazil, 2009-2011.

| **Variables** | **Mortality rates** | | **Mortality ratios (1)/(2)** |
| --- | --- | --- | --- |
|  | **Men^(1)^** | **Women^(2)^** |  |
| **Age** |  |  |  |
| 20-29 | 1.7 | 0.5 | 3.4 |
| 30-39 | 2.3 | 1.0 | 2.4 |
| 40-49 | 4.5 | 1.9 | 2.4 |
| 50-59 | 9.2 | 4.2 | 2.2 |
| Total | 3.9 | 1.7 | 2.3 |
| **Groups of underlying causes (ICD-10)** |  |  |  |
| II. Neoplasms (tumors) | 60.1 | 51.5 | 1.2 |
| IX. Circulatory diseases | 86.7 | 41.6 | 2.1 |
| X. Respiratory diseases | 31.9 | 14.0 | 2.3 |
| XI. Digestive tract diseases | 33.7 | 10.4 | 3.2 |
| XX. External causes | 109.5 | 17.2 | 6.4 |
| **Causes (ICD-BR-10)** |  |  |  |
| Malignant neoplasm of stomach | 5.9 | 2.3 | 2.6 |
| Malignant neoplasm of trachea, bronchus and lungs | 6.9 | 3.2 | 2.1 |
| Diabetes mellitus | 7.3 | 3.3 | 2.2 |
| Hypertensive diseases | 4.6 | 3.0 | 1.5 |
| Acute myocardial infarction | 44.5 | 18.2 | 2.5 |
| Cerebrovascular diseases | 17.5 | 10.1 | 1.7 |
| Pneumonia | 18.6 | 6.0 | 3.1 |
| Liver fibrosis and cirrhosis | 7.9 | 1.2 | 6.7 |
| Transport accidents | 40.3 | 6.1 | 6.6 |
| Homicide | 40.4 | 4.5 | 9.0 |

* deaths per 100,000 inhabitants
